# Extracellular Vesicles Derived from Human Liver Stem Cells Counteract Chronic Kidney Disease Development and Cardiac Dysfunction in Remnant Kidney Murine Model: The Possible Involvement of Proteases

**DOI:** 10.3390/biomedicines12071517

**Published:** 2024-07-08

**Authors:** Elena Ceccotti, Giulia Chiabotto, Massimo Cedrino, Alessandro Gambella, Luisa Delsedime, Alessandra Ghigo, Chiara Salio, Cristina Grange, Maria Beatriz Herrera Sanchez, Saveria Femminò, Marco Sassoè-Pognetto, Maria Felice Brizzi, Giovanni Camussi, Stefania Bruno

**Affiliations:** 1 Department of Medical Sciences, University of Torino, 10126 Torino, Italy; elena.ceccotti@unito.it (E.C.);; 2 Unicyte S.r.l., 10126 Torino, Italy; 3 Pathology Unit, Città delle Salute e della Scienza Hospital, 10126 Torino, Italy; 4 Department of Molecular Biotechnology and Health Sciences, University of Torino, 10126 Torino, Italy; 5 Molecular Biotechnology Center “Guido Tarone”, University of Torino, 10126 Torino, Italy; 6 Department of Veterinary Sciences, University of Torino, Grugliasco, 10095 Torino, Italy; 7 2i3T, Società per la Gestione dell’Incubatore di Imprese e per il Trasferimento Tecnologico, University of Torino, 10126 Torino, Italy; 8 Department of Neurosciences “Rita Levi Montalcini”, University of Torino, 10126 Torino, Italy

**Keywords:** partial nephrectomy, interstitial fibrosis, glomerular sclerosis, diastolic disfunction, matrix metalloproteinase 1

## Abstract

Fibrosis is a marker of chronic kidney disease (CKD) and consists of the accumulation of the extracellular matrix (ECM) components, causing the progressive deterioration of kidney function. Human liver stem cells (HLSCs) have anti-fibrotic activity, and HLSC-derived extracellular vesicles (EVs) mediate this effect. Herein, we evaluated the ability of HLSC-EVs to reverse renal and cardiac alterations in a murine model of partial nephrectomy (PNx) that mimics human CKD development. Furthermore, we investigated the contribution of extracellular matrix remodeling-related proteases to the anti-fibrotic effect of HLSC-EVs. PNx was performed by ligation of both poles of the left kidney, followed one week later by the removal of the right kidney. EV treatment started 4 weeks after the nephrectomy, when renal and cardiac alternations were already established, and mice were sacrificed at week eight. HLSC-EV treatment improved renal function and morphology, significantly decreasing interstitial fibrosis, glomerular sclerosis, and capillary rarefaction. This improvement was confirmed by the decreased expression of pro-fibrotic genes. Moreover, EV treatment improved cardiac function and reduced cardiac fibrosis. HLSC-EVs shuttled different proteases with ECM remodeling activity, and matrix metalloproteinase 1 (MMP-1) was involved in their anti-fibrotic effect on renal tissue. HLSC-EV treatment interferes with CKD development and ameliorates cardiomyopathy in PNx mice.

## 1. Introduction

Chronic kidney disease (CKD) is a worldwide leading cause of mortality and can progress independently from pathogenic triggers as result of a maladaptive processes of injury repair and of hemodynamic alterations due to nephron loss [1]. This process involves the transition of endothelial cells, pericytes, epithelial cells, macrophages, and fibroblasts to myofibroblasts, leading to matrix production. The abnormal synthesis of alpha-smooth muscle actin (alpha-SMA) and collagen subverts the kidney architecture [2]. When kidney function is severely compromised, end-stage renal disease (ESRD) may develop, increasing the risk of a premature death, mainly caused by uremic cardiomyopathy [3]. The actual standard of care for ESRD is only supportive and aims to replace renal function [4]. Although treatments to slow the development of CKD are currently available, the worldwide incidence of CKD and its impact on health care are increasing. For this reason, it is becoming extremely important to develop new therapeutic strategies [1].

Mesenchymal stromal cells (MSCs) are multipotent non-hematopoietic stem cells with anti-fibrotic and immunomodulatory properties able to improve renal damage [5,6,7,8]. The therapeutic potential of MSCs is related to their paracrine activity, which includes extracellular vesicle (EV) release [9]. EVs are small membrane-particles in the nano range involved in cell–cell communication [10]. Their bioactive cargo, which includes proteins, cytokines, growth factors, lipids, mRNAs, and non-coding RNAs, reflects the cell of origin; it is released into the target cell, where it reprograms the molecular pathways involved in disease pathogenesis [11]. EVs derived from different types of stem cells are able to mimic the beneficial effects of the cells of origin in models of tissue injury [12,13,14].

Human liver stem cells (HLSCs), a population of liver-derived cells with mesenchymal-like characteristics, are multipotent, have immunomodulatory capacity, and contribute to tissue regeneration, as shown in several animal models of tissue injury, including renal damage [13,15]. HLSC-derived EVs (HLSC-EVs) mediate the therapeutic properties of the cell of origin and represent a promising cell-free strategy to treat CKD [16,17,18].

Different CKD animal models recapitulate the main pathological alterations occurring in humans. Among them, the 5/6 nephrectomy model mimics the progressive renal failure after the loss of kidney mass in humans [19]. In this study, we tested the effects of HLSC-EV treatment in a murine model of 5/6 partial nephrectomy (PNx) at histological, functional, and molecular levels, both in the remnant kidney and in the heart.

During CKD development, an imbalance in extracellular matrix (ECM) deposition and degradation occurs, and as a result, renal fibrosis is perpetuated [20]. Recent studies highlighted the presence of different matrix metalloproteinases (MMPs) in EVs. It has been reported that EV-associated MMPs can directly contribute to ECM degradation in tumors [21]. Here, we investigated the presence in HLSC-EVs of proteins related to ECM remodeling and evaluated their involvement in the anti-fibrotic effect.

## 2. Materials and Methods

### 2.1. Isolation and Characterization of HLSC-EVs

HLSCs were isolated and characterized as previously reported [15,22,23]. HLSCs were cultured until passage 8. EVs were isolated from the supernatants of sub-confluent HLSCs cultured in HYPERFlasks (Corning, Corning, NY, USA) at a density of 3000 cells/cm^2^ for 18 h in serum-free conditions [18,24,25]. The next day, the cell supernatant was collected, centrifuged at 3000× *g* for 15 min to eliminate cell debris and dead cells, and passed through 0.22 µm filters to exclude apoptotic bodies and large vesicles. EVs were purified by ultracentrifugation at 100,000× *g* for 2 h at 4 °C (Beckman Coulter Optima L-100 K, Fullerton, CA, USA). The pellet was resuspended in RPMI supplemented with 1% dimethyl sulfoxide (DMSO, Sigma-Aldrich, St. Louis, MO, USA) and stored at −80 °C for subsequent studies.

Each EV preparation was quantified using the NanoSight NS300 instrument (NanoSight Ltd., Amesbury, UK) and characterized by cytofluorimetric analysis using a bead-based multiplex analysis system (MACSPlex Exosome Kit, human, Miltenyi Biotec, Bergisch Gladbach, Germany), as reported in previous studies [25,26,27].

To evaluate EV size and integrity, transmission electron microscopy (TEM) was performed as earlier reported [18] and observed using a JEM-1400 Flash transmission electron microscope (JEOL, Tokyo, Japan).

The characterization of HLSC-EVs is shown in Figure S1. HLSC-EVs expressed tetraspanins (CD63, CD81, and CD9) and mesenchymal markers, as described in [18,24,25]. Nanoparticle tracking analyses showed the characteristic size distribution of a heterogeneous population of EVs, and TEM analysis confirmed the presence of an intact membrane.

### 2.2. Protease Array

For protease analysis inside EVs, an iodixanol floating separation protocol was applied to further purify EV preparations from contaminating proteins, as previously described [24,28]. To characterize the expression profile of 35 human proteases in HLSCs and HLSC-EVs, the Proteome Profiler^TM^ Human Protease Array Kit (ARY021B, R&D Systems, Minneapolis, MN, USA) was used, following the manufacturer’s instructions. HLSC and EV lysates (200 µg) were mixed with a cocktail of biotinylated detection antibodies and incubated overnight with Proteome Profiler^TM^ Human Protease Array membranes.

### 2.3. Western Blot Analysis

HLSCs and HLSC-EVs were lysed at 4 °C for 30 min in Lysis Buffer 17 supplemented with 10 µg/mL Aprotinin, 10 µg/mL Leupeptin, and 10 µg/mL Pepstatin (all purchased from R&D Systems, Minneapolis, MN, USA). Protein concentration was assessed using the bicinchoninic acid (BCA) Protein Assay Kit (Pierce™ Thermo Fisher, Waltham, MA, USA), and 5 µg of the protein samples were analyzed as previously described [24]. The following anti-human primary antibodies were used following the manufacturer’s instructions: anti-disintegrin and metalloproteinase domain-containing protein (ADAM) -9 (ADAM9, #4151), anti-MMP1 (#54376), anti-MMP2 (#40994), anti-CD26 (#67138), anti-urokinase-type plasminogen activator (uPA, #15800), anti-CD63 (#52090), anti-CD81 (#56039), anti-CD9 (#13174), and anti-GM130 (#12480), all purchased from Cell Signaling Technology (Danvers, MA, USA).

### 2.4. Partial Nephrectomy 5/6th Murine Model

Animal studies were conducted in accordance with the National Institute of Health Guide for the Care and Use of Laboratory Animals, following the ARRIVE guidelines. The procedures were approved by the Italian Health Ministry (authorization number: 275/2021-PR). Ten-week-old male SCID mice were purchased from ENVIGO (S. Pietro al Natisone, Udine, Italy), weighing from 24 to 26 g, and were anesthetized using an intramuscular (i.m.) injection of zolazepam 80 mg/kg and xylazine 16 mg/kg.

The partial nephrectomy 5/6th (PNx) procedure was performed by a two-step surgery, as described in [19,29]. In step one, the left lumbar area was prepared for surgery by performing a trichotomy followed by disinfection with Iodopovidone (Esoform Manufacturing, Rovigo, Italy). The animal was then placed on the right side on a heating plate to maintain the body temperature at 37 °C. A lumbar incision was performed, and the superior and inferior poles of the left kidney were then ligated using a 4-0 silk suture (Fine Science Tools GmbH, Heidelberg, Germany). In step two, performed 1 week after step one, the right kidney was treated in the same way as the left kidney through the procedure mentioned above. In addition, using angled forceps, a double ligation of the right ureter was carried out before dissecting it. This prevented urine entering the peritoneum from the bladder through the right dissected ureter. To ligate the blood vessels with a 6/0 silk suture (Ethicon, Raritan, NJ, USA), an access channel was created under the artery and the renal vein using angular forceps. Following the correct occlusion of the blood vessels, nephrectomy was performed. After surgery, the muscle and skin were closed with 6-0 silk sutures, and the surgical area was disinfected with Iodopovidone. Afterwards, the mouse was housed in a designated cage under a heat lamp at 37 °C for 20 min.

**Figure 1:**
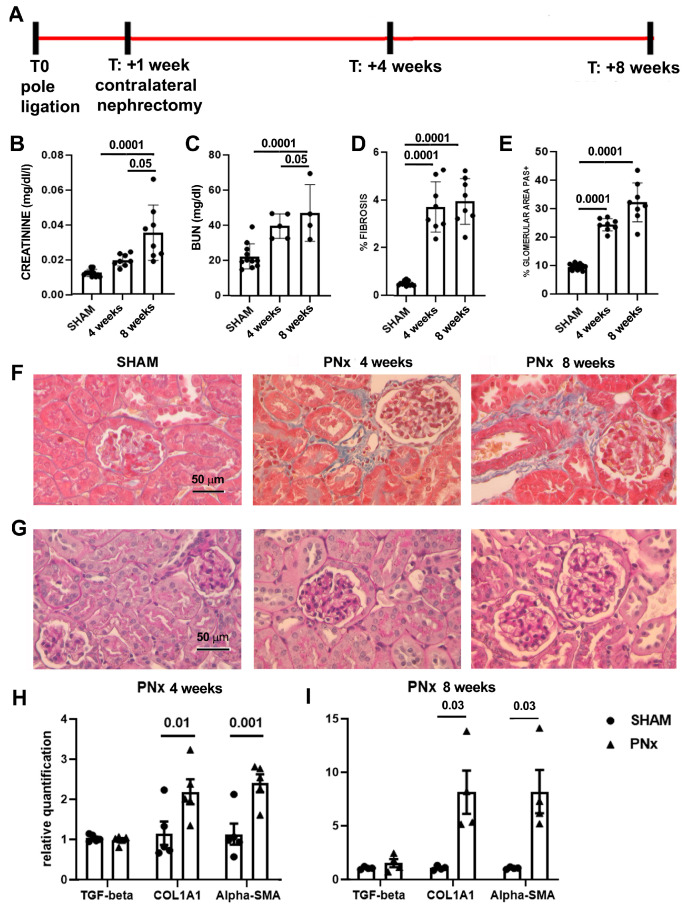
CKD development in 5/6th partial nephrectomy (PNx) model. (**A**) Layout of the experiments to set up the in vivo model. At T0, mice were subjected to pole ligation of the left kidney; one week later, the right kidney was removed (contralateral nephrectomy). Mice were sacrificed 4 and 8 weeks after the second surgery to evaluate CKD progression. (**B**,**C**) Evaluation of creatinine (**B**) and BUN (**C**) plasma levels of control SHAM mice (n = 12) and PNx mice sacrificed 4 and 8 weeks after nephrectomy (n = 8 mice/time point). Results are shown as mean ± SD. (**D**) Quantification of fibrosis by histological analyses in SHAM and PNx mice sacrificed 4 and 8 weeks after nephrectomy. Results are shown as mean ± SD. (**E**) Histological quantification of glomerular PAS+ deposition in SHAM and PNx mice sacrificed 4 and 8 weeks after nephrectomy. Results are shown as mean ± SD. (**F**) Representative photographs of Masson’s trichrome stained renal sections of SHAM and PNx mice sacrificed 4 and 8 weeks after nephrectomy. The blue stain represents collagen fibers. Original magnification: 400×. Bar scale: 50 μm. (**G**) Representative micrographs of PAS-stained renal sections of SHAM and PNx mice sacrificed 4 and 8 weeks after nephrectomy. Original magnification: 400×. Bar scale: 50 μm. (**H**,**I**) Gene expression levels of fibrotic markers (*TGF-beta*, *COL1A1*, *and alpha-SMA*) in PNx mice sacrificed 4 ((**H**), n = 5) and 8 ((**I**), n = 4) weeks after nephrectomy with respect to SHAM mice. Data are normalized to *GAPDH*. Mean ± SEM was calculated by comparing the gene expression levels of each group with the ones of the SHAM mice.

A control group of mice (SHAM) underwent the same surgical procedure without ligation of the poles of the left kidney and without nephrectomy of the right kidney. To set up the experimental protocol, PNx mice were sacrificed 4 and 8 weeks (n = 8/experimental point) after nephrectomy (Figure 1).

To evaluate the effect of EVs, 4 weeks after nephrectomy, PNx mice were randomly divided and treated with the vehicle alone (n = 10) or with different amounts of EVs, once a week for 4 weeks. Different doses of EVs [18,25] were administered: 4 × 10^9^ EV/mice/administration (dose 1, n = 10), 1 × 10^9^ EV/mice/administration (dose 2, n = 6), and 1 × 10^8^ EV/mice/administration (dose 3, n = 6). A group of PNx mice (n = 6) received dose 1, pretreated with neutralizing anti-human MMP1 antibody (MAB901, 200 μg/mL, R&D Systems). Mice were sacrificed 8 weeks after nephrectomy, and blood, remnant kidney, and heart were recovered for analyses.

### 2.5. Renal Functional and Histological Analysis

Renal function was assessed by biochemical analyses, as previously reported [18]. Kidney histology was investigated in tissue fixed in formalin and paraffin-embedded. Paraffin sections were stained with Masson’s trichrome (Bio-Optica, Milan, Italy) or Periodic Acid Schiff (PAS, Bio-Optica) reaction according to the manufacturer’s instructions.

The surface area occupied by collagen was quantified in non-overlapping high-power field (HPF) (n = 10/section) analyzing photographs (original magnification: 400×). ImageJ software (version 1.49s) was utilized to perform multiphase image analyses.

The glomerular deposition of the PAS-positive ECM was determined by ImageJ software on 15 glomeruli from each mouse at a magnification of 400×.

The expression of the endothelial marker CD31 was evaluated on renal sections from paraffin-embedded blocks, using an anti-mouse CD31 antibody (#3528, Cell Signaling Technology). Hydrogen peroxidase (6%) was applied for 8 min at room temperature (RT) to block the activity of endogenous peroxidase. After this, antigen retrieval was performed by boiling in ethylenediamine tetra-acetic acid buffer (pH 9) for 30 min. The primary antibody (1:100) was incubated overnight at 4 °C. The omission of the primary antibody was used as a negative control. Incubation with horseradish peroxidase-labeled anti-goat antibody (Thermo Fisher Scientific) was performed for 1 h at RT. 3,3-diaminobenzidine was used to develop the reaction product.

### 2.6. Transmission Electron Microscopy (TEM)

Kidneys of PNx mice treated with the vehicle alone (n = 3) or with dose 1 (n = 3) and of SHAM mice (n = 3), sacrificed 4 weeks after nephrectomy, were rapidly dissected and cut in small pieces (1–2 mm thick), which were fixed by immersion in phosphate buffer (0.1 M, pH 7.4) containing 2.5% glutaraldehyde for 48 h at 4 °C. Post-fixation in osmium ferrocyanide (1:1 volume ratio of 2% aqueous osmium tetroxide and 3% potassium ferrocyanide) was carried out for 1 h at 4 °C, followed by dehydration for 15 min in increasing concentrations of acetone (30–60–90 and 100%) and incubation in Spurr resin diluted in acetone 100% (1:1 volume ratio for 30 min; 2:1 volume ratio for 30 min) and in Spurr resin (Electron Microscopy Sciences, Hatfield, PA, USA) overnight at RT. As a last step, embedding in Spurr resin was performed for 24 h at 70 °C. Ultramicrotome (EM UC6, Leica Microsystems, Wetzlar, Germany) was used to cut ultrathin sections, which were collected on uncoated nickel grids (100 mesh) and counterstained for 30 s with UranyLess EM Stain and 30 s with lead citrate (Electron Microscopy Sciences). Sections were observed with a JEM-1400 Flash transmission electron microscope, and photographs were acquired with a high-sensitivity sCMOS camera.

### 2.7. Molecular Analysis of Renal Tissue

The RNA was extracted from the renal tissue of the SHAM or PNx mice treated or not with dose 1 and doses 2 of HLSC-EVs using TRIzol™ reagent (Ambion, Thermofisher, Waltham, MA, USA), as previously described [22]. Complementary DNA (cDNA) was retrotranscribed from murine RNA by a High-Capacity cDNA Reverse Transcription Kit (Applied Biosystems, Foster City, CA, USA). To evaluate specific gene expression by quantitative Real-Time PCR (qRT-PCR), a 96-well QuantStudio^TM^ 12K Flex Real-Time PCR system (Thermo Fisher Scientific) was used as previously reported [22]. The primers used for qRT-PCR are listed in Table 1.

For all samples, fold-change expression with respect to the PNx group or the SHAM group was calculated using the ΔΔCt method.

### 2.8. Cardiac Histological Analyses

Structural damage to the heart was assessed through histopathological analysis. Two pathologists evaluated and reported histopathological (e.g., atrophy, inflammation, necrosis, and fibrosis) characteristics.

Pathological features were recorded and graded based on the extent (none, mild, moderate, of severe), distribution (focal, multifocal, or diffuse), and subsite location (epicardium, myocardium, of endocardium) [30,31,32]. Cardiomyocyte atrophy was defined as cell size decrease. In addition, as the heart is considered an immune “sanctuary”, the presence of any inflammatory cells within the cardiomyocytes was considered pathological and was recorded. Cardiomyocytes with cytoplasmic eosinophilia and nuclear loss accompanied by interstitial edema or loss of myocardial fibers were considered for necrosis assessment and graded. Finally, cardiac fibrosis was reported and further differentiated in interstitial (fibrotic bundles outlining cardiomyocytes), replacement/scarring (fibrotic deposits replacing injured/necrotic cardiomyocytes), and perivascular (fibrotic tissue surrounding capillaries and small vessels) fibrosis [33,34].

### 2.9. Transthoracic Echocardiography

Mice were anesthetized with 1% isoflurane and analyzed with a Vevo 2100 High Resolution Imaging System (Visual Sonics Inc., Toronto, ON, Canada) equipped with a 30 MHz probe (MS550D) (Visual Sonics). Left ventricle mass (LV mass), left ventricle internal diameters (LVID), and interventricular septum thickness (IVS) were measured at the level of the papillary muscles in the parasternal short-axis view (M mode). Mitral valve deceleration time was measured with tissue Doppler and pulsed wave Doppler techniques in the apical long-axis view. All measurements were averaged on 3 consecutive cardiac cycles per experiment, and cardiac function was assessed when the heart rate was 400–450 bpm. LV mass parameter was normalized to body weight.

### 2.10. Statistical Analyses

Statistical analysis was performed using GraphPad Prism software version 8.0 (GraphPad Software, Inc., La Jolla, CA, USA). All data are shown as mean ± SD or SEM. Comparison among control groups and the different experimental groups were performed using One-Way ANOVA followed by Tukey’s multiple-comparison test or Two-Way ANOVA followed by Sidak’s or Dunnett’s multiple-comparison tests. A *p*-value < 0.05 was considered significant.

## 3. Results

### 3.1. Set-Up of the In Vivo Model

Biochemical analyses of markers of renal dysfunction found a significant increment in creatinine and blood urea nitrogen (BUN) plasma levels in mice subjected to PNx and sacrificed 4 weeks after nephrectomy (Figure 1). PNx mice sacrificed at week 8 had a further significant increase in BUN and creatinine, indicating the worsening of the kidney function.

At the histological level, PNx mice sacrificed 4 and 8 weeks after the nephrectomy showed signs of CKD development. Specifically, the presence of interstitial fibrosis was observed at both time points, and quantification of the fibrotic area indicated that a significant increase in fibrosis was already detectable at week 4 (Figure 1). At the glomerular level, we observed an increase in the glomerular deposition of PAS-positive ECM, suggestive of glomerulosclerosis (Figure 1).

Molecular analysis of genes involved in the development of fibrosis demonstrated that PNx mice exhibited a significant increase in the expression levels of alpha-SMA and collagen I (COL1A1) (Figure 1).

### 3.2. EV Administration Improves Kidney Function and Morphology

**Figure 2:**
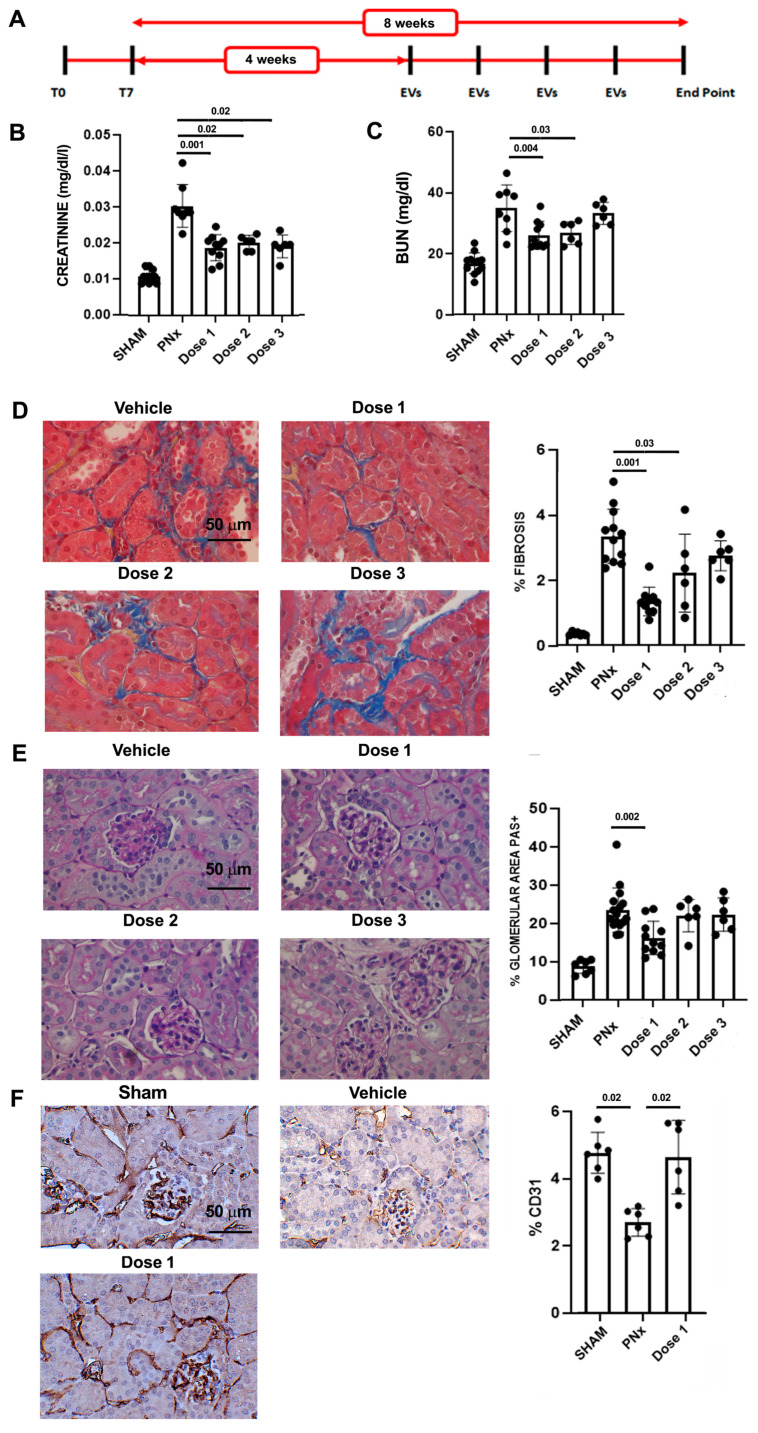
Effects of different EV doses on renal function and morphology. (**A**) Experimental layout to test EVs in CKD mice, showing the time points of surgeries (T0 and T7), EV administrations, and sacrifice (end point). (**B**,**C**) Evaluation of creatinine (**B**) and BUN (**C**) plasma levels of control SHAM mice (n = 12) and PNx mice sacrificed 8 weeks after surgery treated with vehicle alone (n = 8) or with different doses of EVs (n = 10/dose 1 and n = 6/doses 2 and 3). Results are shown as mean ± SD. (**D**) Representative photographs of Masson’s trichrome stained renal sections of PNx mice treated with vehicle alone or with different doses of EVs. Original magnification: 400×. Bar scale: 50 μm. Bar chart on the right represents quantification of fibrosis of the different experimental groups. (**E**) Representative images of PAS-stained renal sections of PNx mice treated with vehicle alone or with different doses of EVs. Original magnification: 400×. Bar scale: 50 μm. Bar chart on the right represents histological quantification of glomerular PAS+ deposition in the different experimental groups. (**F**) Representative photographs of CD31 antibody-stained renal sections of SHAM mice and PNx mice treated with vehicle alone or with dose 1. Original magnification: 400×. Bar scale: 50 μm. In the graph on the right, CD31 expression was quantified in each experimental group.

We evaluated whether the administration of HLSC-EVs revert functional and histopathological alterations in PNx mice. EV treatment started 4 weeks after the nephrectomy, when the histopathological signs of CKD development were evident (Figure 2).

EV treatment improved renal function: in fact, all tested doses significantly reduced creatinine plasma level (Figure 2) with respect to PNx mice treated with the vehicle alone. The reduction in BUN reached statistical significance in PNx mice treated with doses 1 and 2 (Figure 2).

Assessment of histological alterations indicated that EV treatment with dose 1 significantly reduced both interstitial fibrosis and glomerulosclerosis (Figure 2). Likewise, dose 2 significantly reduced interstitial fibrosis but did not ameliorate glomerular sclerosis (Figure 2).

Another important feature of CKD is capillary rarefaction. EV administration significantly increased the percentage of CD31-positive cells in PNx mice, indicating that EVs are effective in counteracting capillary rarefaction (Figure 2).

**Figure 3:**
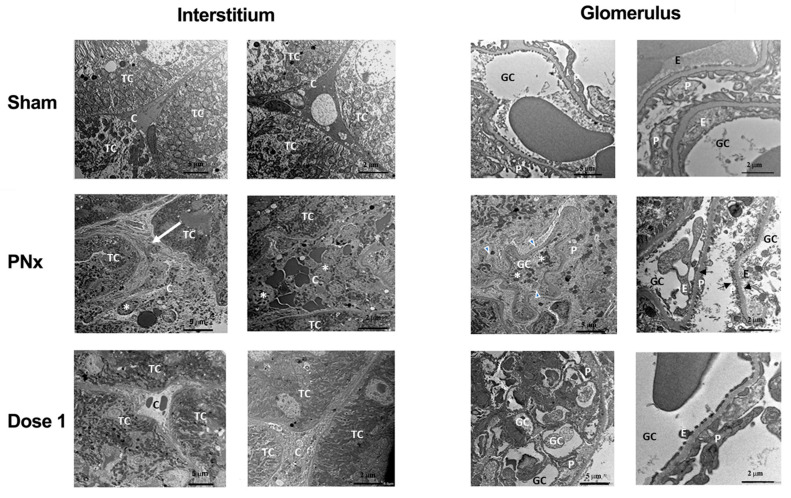
Ultrastructural changes of interstitium and glomeruli in PNx mice and effects of EV treatment. TEM representative images of interstitium and glomerulus of SHAM and PNx mice treated with vehicle alone or with dose 1, sacrificed 8 weeks after nephrectomy. Vehicle-treated PNx mice show increased ECM accumulation and monocyte infiltration with loss of peritubular capillaries, as well as glomerular alterations characterized by endothelial and epithelial injuries and effacement of podocytes. These alterations were less evident in sections from PNx mice treated with EVs (dose 1). Scale bars indicate the magnification. Abbreviation and symbols: TC = tubular cells; C = peritubular capillaries; * = monocytes and leukocytes; white arrow = extracellular matrix accumulation; GC = glomerular capillaries; P = podocytes; E = glomerular endothelial cells; head arrows = podocyte effacement; black arrows = necrotic podocytes and endothelial cells.

Similar alterations were also observed at the ultrastructural level (Figure 3). Vehicle-treated PNx mice showed accumulation of ECM components and monocyte infiltration in peritubular regions due to loss of peritubular capillaries. Moreover, there were evident signs of endothelial and epithelial suffering in glomeruli. These lesions were markedly reduced after EV treatment. In particular, the enlargement and infiltration of interstitium, the loss of endothelium and effacement of podocytes and glomeruli were reduced (Figure 3).

### 3.3. EV Treatment Modulates the Expression of Fibrosis and Inflammation-Related Genes

**Figure 4:**
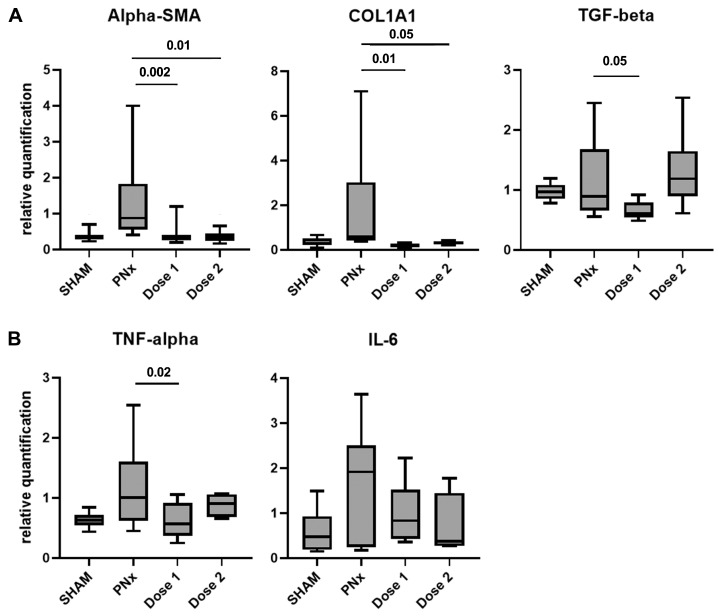
Expression levels of pro-fibrotic and pro-inflammatory genes in PNx mice treated with EVs or with vehicle alone. Gene expression levels of the fibrosis-related (*alpha-SMA*, *COL1A1*, and *TGF-beta*) (**A**) and inflammatory markers (*TNF-alpha* and *IL-6*) (**B**) in PNx mice treated with doses 1 (n = 10) and 2 (n = 6) of EVs or with vehicle alone (n = 10) and SHAM mice (n = 10). Data are normalized to GAPDH. Mean ± SEM was calculated by comparing the gene expression levels of each experimental group with those of the PNx group treated with the vehicle alone.

PNx mice treated with doses 1 and 2 showed a significant reduction in *alpha-SMA* and *COL1A1* gene expression levels compared to PNx mice treated with the vehicle alone. The gene expression level of *TGF-beta* was significantly down-regulated only in PNx mice treated with dose 1 (Figure 4). Furthermore, a significant increase in tumor necrosis factor (*TNF*)*-alpha* and interleukin (*IL*)*-6* gene expression levels was detected in renal tissue of PNx mice (Figure 4). A reduction in the expression of these two inflammatory markers was observed only in PNx mice treated with dose 1 (Figure 4).

### 3.4. EV Treatment Ameliorates Cardiac Function and Morphology

Echocardiographic analysis was performed to evaluate the effects of PNx surgery and of HLSC-EV administrations on cardiac function.

**Figure 5:**
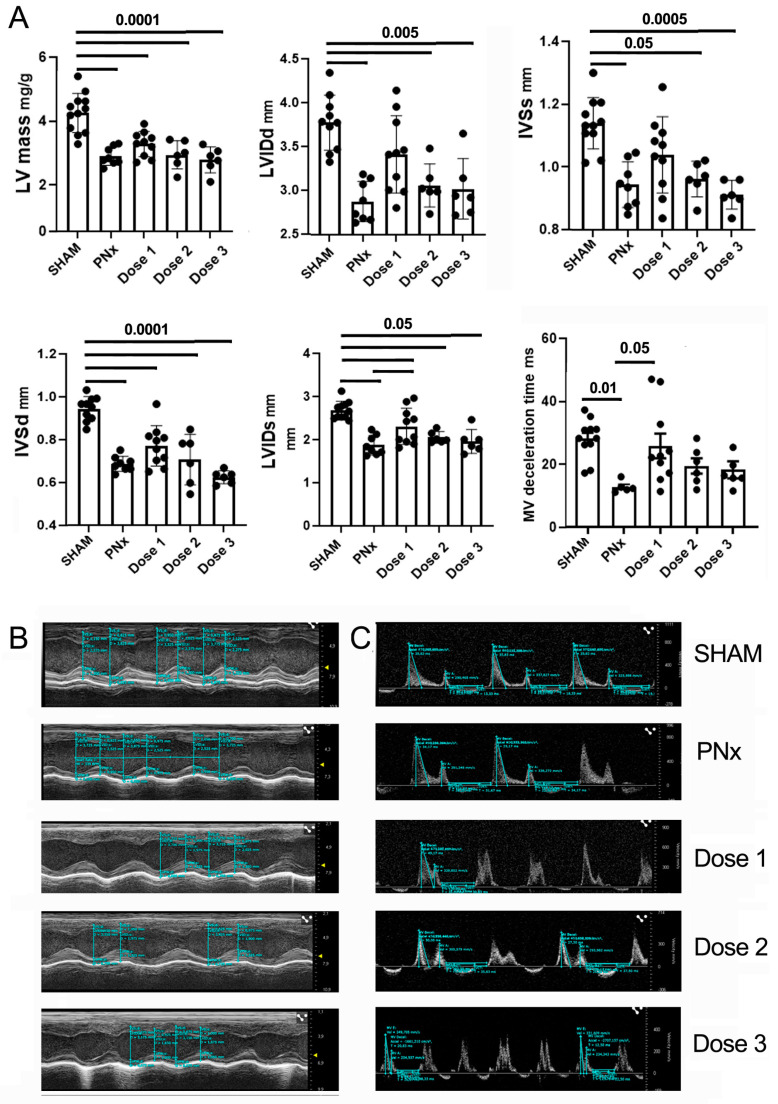
Effects of different EV doses on cardiac function. (**A**) Bar charts showing the results of echocardiographic analysis of SHAM mice and PNx mice of different experimental groups. Results are shown as mean ± SD. (**B**,**C**) Representative echocardiographic images of SHAM and PNx mice, treated or not with different EV doses. In panel (**B**), M-mode measurements are showed to obtain LV mass and for the assessment of systolic function (LVIDd, IVSs, IVSd, and LVIDs). In panel (**C**), doppler analyses of the mitral valve are shown, to obtain MV deceleration time.

Compared to SHAM mice, PNx mice injected with the vehicle showed a reduction in LV mass and myocardial wall thickness, in particular at the level of the IVS, as well as a reduction of the LVID (Figure 5). Furthermore, a reduction in mitral valve (MV) deceleration time, a hallmark of diastolic dysfunction, was observed. After treatment of PNx mice with EV dose 1, there was a trend towards amelioration for several parameters (LV, IVS, LVID), with a statistically significant recovery for LVIDs and MV deceleration time (Figure 5).

**Figure 6:**
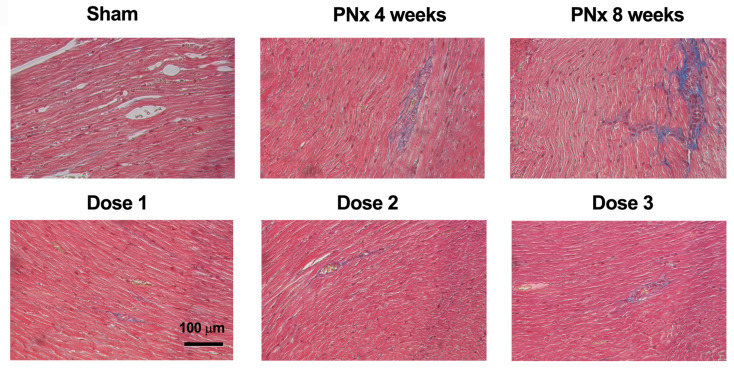
Effects of different EV doses on cardiac morphology. Photographs of cardiac sections stained with Masson’s trichrome of representative SHAM and PNx mice sacrificed 4 weeks after nephrectomy and PNx mice sacrificed 8 weeks after nephrectomy and treated with vehicle alone or with different doses of EVs. The blue stain represents collagen fibers. Original magnification: 200×. Bar scale: 100 μm.

Interstitial fibrosis is considered a key hallmark of diastolic dysfunction. Histopathological analysis revealed the presence of a mild to moderate focal or multifocal interstitial fibrosis in the sub-endocardial area of all PNx mice sacrificed 4 and 8 weeks after nephrectomy and in 6 out of 7 vehicle-treated PNx mice. Notably, no signs of interstitial fibrosis were observed in most of the PNx mice treated with EVs (Figure 6 and Table 2). None of the mice presented cardiomyocyte atrophy, inflammation, or necrosis.

These data indicate that the EV treatment had an anti-fibrotic effect on cardiac tissue.

### 3.5. Protease Content of EVs and Its Implication for Their Anti-Fibrotic Effect In Vivo

**Figure 7:**
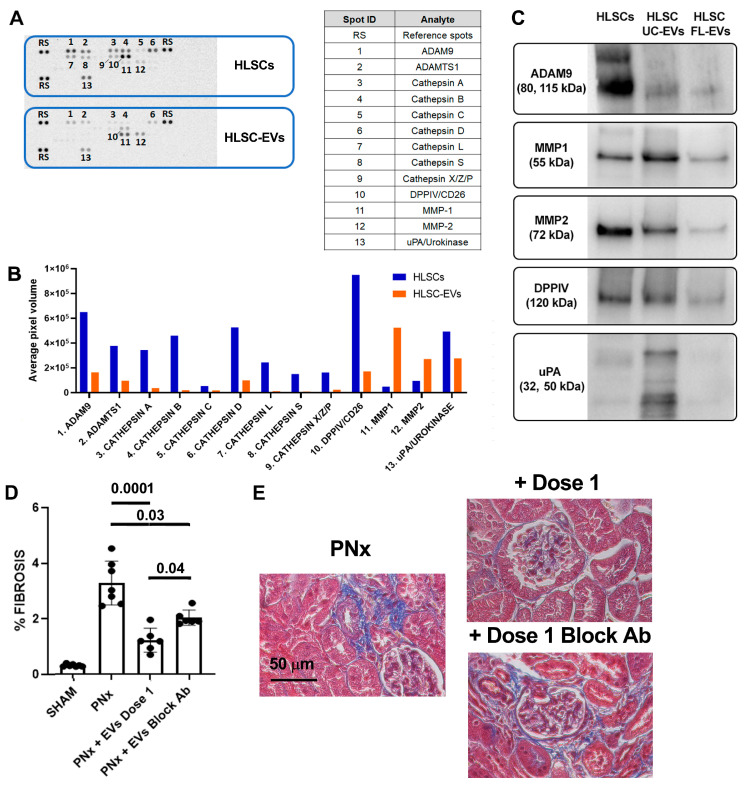
Protease expression profile in EVs. (**A**) Representative image of the results of Proteome Profiler^TM^ Human Protease Array, comparing the expression of 35 human proteases among HLSC and HLSC-EV protein lysates. The top 13 proteases expressed in cells and EVs are listed in the table on the right. (**B**) Histogram representing the average signal (pixel density of the spots) of the 13 proteases expressed in cell protein lysate (blue bars) and in EV lysate (orange bars). (**C**) Representative image of the results of Western blot analyses of 5 proteases expressed in the protein lysate of cells and EVs purified by ultracentrifugation (UC-EVs) and EVs purified by floating (FL-EVs). (**D**) Histological quantification of fibrosis in SHAM mice, PNx mice treated with vehicle alone, and PNx mice injected with dose 1 of EVs pre-treated or not with blocking antibody (n = 6/group). Results are shown as mean ± SD. (**E**) Representative images of renal sections of PNx mice treated with vehicle alone or with dose 1 of EVs pre-treated or not with specific blocking antibody stained with Masson’s trichrome. The blue stain represents collagen fibers. Original magnification: 400×. Bar scale: 50 μm.

Proteomic analyses using an antibody-based array approach showed an EV profile related to the ECM remodeling capacity (Figure 7).

The screening of 35 proteases demonstrated that, compared to the cells of origin, EVs are enriched in MMP1 and 2. In both cells and EVs, other proteases with ECM degradation activities were detected, such as ADAM-9, ADAM with thrombospondin motifs 1 (ADAMTS1), dipeptidyl peptidase-4 (DPPIV, also known as CD26), and urokinase (uPA) (Figure 7). Interestingly, the protein expression of MMP1 and 2, ADAM9, and DPPIV, in both EVs isolated by ultracentrifugation (UC-EVs) and EVs isolated by floating (FL-EVs) indicated that these proteases are shuttled by EVs and not only co-precipitated during the ultracentrifugation procedure (Figure 7).

To investigate the involvement of the proteases transported by EVs in their in vivo anti-fibrotic effect, a neutralizing anti-human MMP1 antibody was used to treat EVs before their in vivo administration. Treatment of EVs with this neutralizing antibody decreased their in vivo beneficial effect (Figure 7). In particular, in PNx mice that received EVs pre-treated with the MMP1-blocking antibody, a decreased capacity of EVs to reduce renal fibrosis was observed at the histological level (Figure 7). Pre-treatment of EVs with anti-human MMP1 antibody did not influence their anti-fibrotic effect on cardiac tissue.

## 4. Discussion

PNx is a widely applied CKD model, which recapitulates the main features of disease development after loss of kidney mass and function in humans. The conventional subtotal nephrectomy, the so-called ablation model, includes the removal of one kidney and the excision of two-thirds of the other kidney. In this model, it was demonstrated that vesicles obtained from bone marrow (BM)-derived MSCs protected the kidney from injury. Like BM-MSCs, BM-MSC-EVs reduced proteinuria, interstitial fibrosis, lymphocyte infiltrates, and tubular atrophy [35]. Similar results were obtained in a rat model of 5/6th ablation, where BM-MSC-EVs protected against kidney injury by upregulating the expression and the activity of klotho, a reno-protective molecule [36]. The conventional ablation model may cause post-operative bleeding, infection, and mortality [19]. A new surgical procedure was recently introduced, which consists of a two-step surgery: at first, the upper and lower poles of the left kidney are ligated; then, a week later, the right kidney is removed (nephrectomy). One month after the nephrectomy, PNx animals develop the common hallmarks of CKD: interstitial fibrosis, glomerular sclerosis, and uremic cardiomyopathy [29].

We established the PNx ligation model in immunodeficient SCID male mice. In this murine model, we tested the therapeutic effects of HLSC-EVs on the development of CKD, cardiac fibrosis, and diastolic dysfunction.

Our data showed that HLSC-EVs have favorable effects in different organs at both functional and histological levels, as previously reported for EVs isolated from adipose-derived MSCs [37]. In fact, we demonstrated that multiple HLSC-EV administrations exerted beneficial effects on renal function and morphology, both at the tubular and glomerular levels. Furthermore, the results obtained on cardiac tissue reveal for the first time that HLSC-EVs also exert a beneficial effect on cardiac fibrosis and diastolic dysfunction. Based on this evidence, we can speculate a direct anti-fibrotic effect of HLSC-EVs on the heart, but we cannot exclude an indirect beneficial effect due to renal amelioration.

In renal tissues of PNx mice treated with HLSC-EVs, we observed down-regulation of gene expression levels of specific markers of fibrosis, such as alpha-SMA, COL1A1, and TGF-beta. Similar results have been previously obtained by injecting HLSC-EVs in mouse models of aristolochic acid-induced kidney fibrosis [16] and in ischemia and reperfusion-CKD [18]. In a murine model of diabetic nephropathy (DN), [17] microRNAs (miRNAs) shuttled by HLSC-EVs showed anti-fibrotic and anti-inflammatory effects. The miRNA cargo of HLSC-EVs includes miR-17-5p, miR-106a-5p, and miR-155-5p, which are involved in the regulation of different molecular pathways, or adhesion molecule-cadherin signaling pathways, known to contribute to fibrosis progression. Moreover, HLSC-EVs shuttle miR-146a, which contributes to the inhibition of inflammation in DN-CKD [17,38,39] and also to the in vitro anti-fibrotic activity of HLSC-EVs [24].

The biological activity of HLSC-EVs is also influenced by their protein content. In fact, our previous proteomic analysis revealed the presence of several anti-inflammatory proteins in the HLSC-EV cargo [25]. Most of them are involved in IL-10, p53, or PI3K signaling pathways, thus contributing to the HLSC-EV anti-fibrotic and anti-inflammatory activity. To better understand the mechanisms associated with the beneficial effects of HLSC-EVs, we analyzed the protease content of our vesicle population. Protein array and Western blot analyses showed that HLSC-EVs shuttled MMP1, MMP2, and other proteases with ECM degradation capacity. Similarly, using mass spectrometry and an antibody array, it has been reported that exosomes purified from embryonic stem cell (ESC)-derived MSCs contain MMP1, MMP3, MMP10, ADAM9, and ADAMTS12 [40]. However, the possible involvement of these proteases in the in vivo beneficial effects of ESC-MSC-EVs has not been evaluated. Here, we report the possible contribution of proteases associated with HLSC-EVs to their anti-fibrotic activities in renal tissue. In particular, by administering HLSC-EVs pre-treated with specific anti-MMP1 blocking antibody to PNx mice, we observed a reduced capacity of these EVs to counteract fibrosis development in the kidney but not in the heart. These data indicate a possible implication of MMP1 in the therapeutic potential of HLSC-EVs, at least in renal tissue.

Recently, an anti-fibrotic activity of MMP1-decorated polymersomes (MMPsomes) has been reported in an in vivo model of hepatic fibrosis [41]. This innovative approach of MMP1 delivery, using surface-decorated synthetic vesicles, seems to be efficient in reducing collagen deposition in vivo. HLSC-EVs naturally transport MMP1 and other ECM-degrading proteases. Therefore, collagen degradation capacity is a candidate mechanism underlying the anti-fibrotic effect of HLSC-EVs that could involve proteases and other EV components (e.g., miRNA, mRNA, etc.) in reducing tissue fibrosis

## 5. Conclusions

In conclusion, our results show that HLSC-EVs, administered in multiple doses in PNx mice with established CKD and cardiac dysfunction, can effectively improve renal and cardiac function and morphology, mainly through the reduction of tissue fibrosis. Moreover, we demonstrated that proteases shuttled by HLSC-EVs could have a role in their in vivo anti-fibrotic activity in renal tissue. In particular, EV-mediated delivery of MMP1 seems to be implicated, since specific blocking of MMP1 decreases the ability of EVs to attenuate fibrosis in the renal tissue of PNx mice. Further studies will be necessary to better elucidate the mechanism of action of the proteases shuttled by EVs in their in vivo anti-fibrotic activity.

## Figures and Tables

**Table 1:** Primer pairs used for the detection of murine genes by qRT-PCR.

Gene Name	Forward (5′-3′)	Reverse (5′-3′)
*GAPDH*	TGTCAAGCTCATTTCCTGGTA	TCTTACTCCTTGGAGGCCATGT
*Alpha-SMA*	CATCTCCGAAGTCCAGCACA	GACGCACCACTGAACCCTAA
*COL1A1*	ACCTTGTTTGCCAGGTTCAC	ATCTCCCTGGTGCTGATGGAC
*IL-6*	ACCAGAGGAAATTTTCAATAGGC	TGATGCACTTGCAGAAAACA
*TGF-beta*	GCAACAATTCCTGGCGTTACC	CGAAAGCCCTGTATTCCGTCT
*TNF-alpha*	CATCTTCTCAAAATTCGAGTGACAA	TGGGAGTAGACAAGGTACAACCC

**Table 2:** Histopathological results of cardiac tissue analyses.

Group	Fibrosis				
	Type	Number of Cases with Increased Fibrosis	Overall Grade	Location	Distribution
SHAM	Absent	0/8	None		
PNx week 4	Interstitial	3/3	Mild	Sub-endocardium	Focal
PNx week 8	Interstitial	5/5	Mild	Sub-endocardium	Multifocal
PNx + vehicle	Interstitial	6/7	Mild	Sub-endocardium	Focal
PNx + EVs Dose 1	Interstitial	1/10	Mild	Sub-endocardium	Focal
PNx + EVs Dose 2	Interstitial	1/6	Mild	Sub-endocardium	Focal
PNx + EVs Dose 3	Interstitial	1/6	Moderate	Sub-endocardium	Focal

## Data Availability

The original contributions presented in the study are included in the article/Supplementary Material; further inquiries can be directed to the corresponding author/s.

## Supplementary Materials

The following supporting information can be downloaded at: https://www.mdpi.com/article/10.3390/biomedicines12071517/s1, Figure S1: EV characterization.
