# Supplementary material for: Extracellular Vesicles Derived from Human Liver Stem Cells Counteract Chronic Kidney Disease Development and Cardiac Dysfunction in Remnant Kidney Murine Model: The Possible Involvement of Proteases
Source: Biomedicines. 2024 Jul 8;12(7):1517. doi: 10.3390/biomedicines12071517 (PMC11274379; doi:10.3390/biomedicines12071517)
Supplement: Supplementary file 1 [file biomedicines-12-01517-s001.zip › biomedicines-3048465-supplementary.pdf]

Supplementary Materials

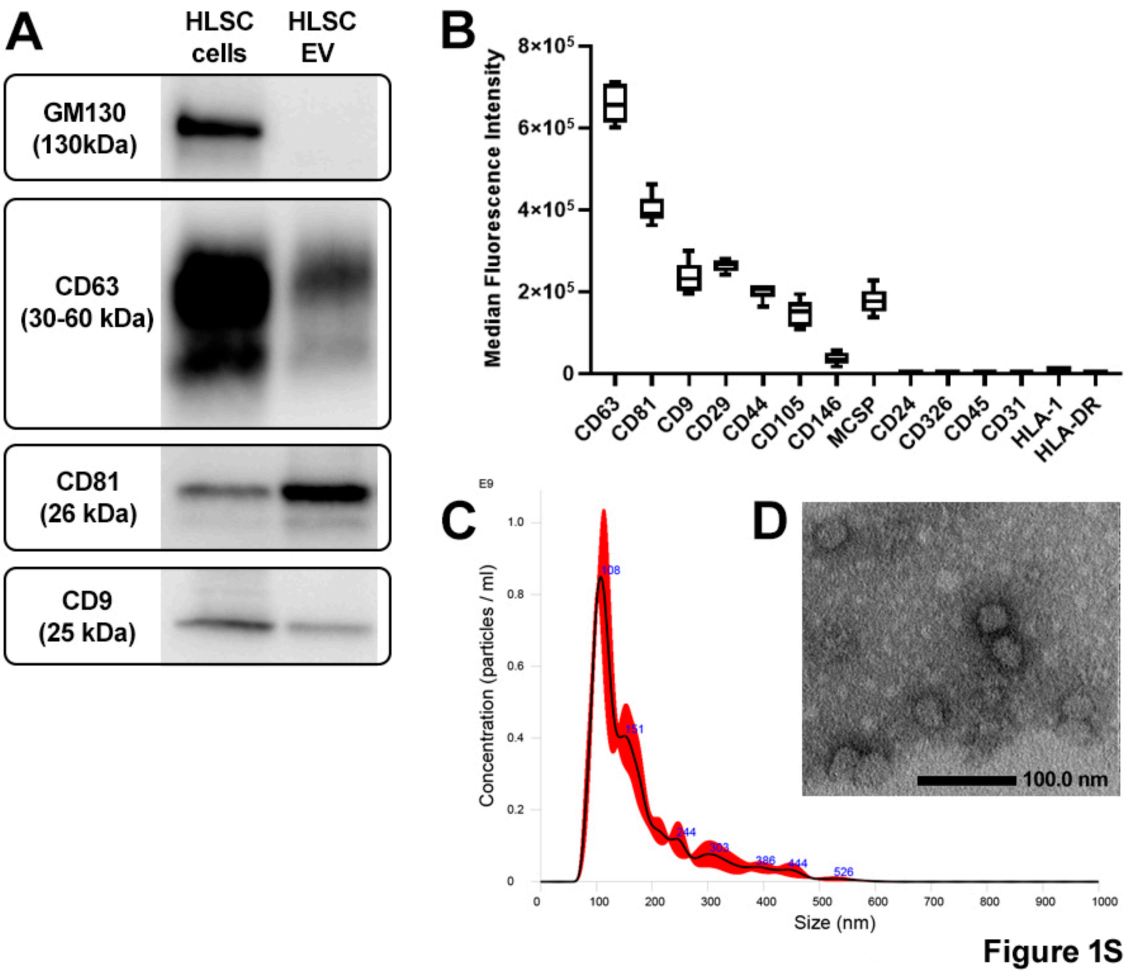

Supplementary Figure S1. EV characterization. A) Representative Western showing the expression of the tetraspanins CD63, CD81 and CD9 in both HLSCs and HLSC-EV. As expected, only HLSC expressed the *cis*-Golgi protein GM130, and EVs. **B)** Cytofluorimetric analysis of surface proteins expressed on EVs. The graph shows the median allophycocyanin fluorescence values of 14 bead populations selected from 37 that can be screened using the MACSPlex Exosome Kit for bead-based multiplex analysis. Measurement of the median fluorescence intensity (MFI) was performed using the CytExpert Software on three different EV preparations. In order to remove background fluorescence, the MFI value of a blank control (MACSPlex buffer + capture beads + detection antibodies) was subtracted from the MFI value of each capture bead subset. **C)** Representative NTA chart illustrating concentration and size distribution of EVs. **D)** TEM figure of EVs contrasted with Nano-W™ and NanoVan™ (scale bar, 100 nm).
